# Supplementary material for: MetaRibo-Seq measures translation in microbiomes
Source: Nat Commun. 2020 Jun 29;11:3268. doi: 10.1038/s41467-020-17081-z (PMC7324362; doi:10.1038/s41467-020-17081-z)
Supplement: Supplementary file 10 — Supplementary Data 7 [file 41467_2020_17081_MOESM10_ESM.zip › File2/Confidence_High_Taxonomy/396299_out.krona.html]

Javascript must be enabled to view this page.

members
magnitude
magnitudeUnassigned
count
unassigned
taxon
rank

396299\_out

3

superkingdom
3
2

3
201174
phylum

class
3
1760

85004
3
order

family
31953
3

1
genus
1678
3

SRS144362\_contig\_number\_16381


SRS014736\_contig\_number\_6886SRS147039\_contig\_number\_contig-100\_7659.7660
species
216816
2
